# Low 25-hydroxyvitamin D levels are more prevalent in Canadians of South Asian than European ancestry inhabiting the National Capital Region of Canada

**DOI:** 10.1371/journal.pone.0207429

**Published:** 2018-12-12

**Authors:** Reiko Nagasaka, Eleonora Swist, Kurtis Sarafin, Claude Gagnon, Isabelle Rondeau, Isabelle Massarelli, Winnie Cheung, Patrick Laffey, Stephen PJ Brooks, W. M. Nimal Ratnayake

**Affiliations:** 1 Bureau of Nutritional Sciences, Health Products and Food Branch, Health Canada, Ottawa, ON, Canada; 2 Bureau of Food Surveillance and Science Integration, Health Products and Food Branch, Health Canada, Ottawa, ON, Canada; Charles P. Darby Children's Research Institute, UNITED STATES

## Abstract

The US Institute of Medicine defined serum 25-hydroxyvitamin D (25OHD) cut point values of 30 nmol/L and 40 nmol/L were used to assess the vitamin D status of South Asian and European Canadians of self-identified ancestry living in the National Capital Region of Canada. Serum 25OHD values were measured in the spring and fall of 2012 to represent status during the winter and summer months, respectively. A total of 1238 measurements were obtained from 669 participants (49% South Asian ancestry): some participants were measured only once (spring or fall). Median 25OHD values were significantly higher in participants of European ancestry: 70.8 nmol/L (68.1, 73.5; 95% CI) versus South Asian ancestry: 42.7 nmol/L (40.5, 45.0; P<0.001). Spring vs. fall differences were small for each ethnic group and significant only for those of European ancestry (2.9, CI: 1.0–4.9 nmol/L; P = 0.01). Among participants of South Asian ancestry, 27.3% (fall) and 29.1% (spring) of females had values <40 nmol/L while the percentages for males were considerably higher (36.5% and 44.2%, respectively). The corresponding values for participants of European ancestry were ≤10%, showing that the South Asian participants were less likely to achieve the 25OHD concentrations established by the IOM for optimum bone health. Investigation of the factors related to serum 25OHD levels showed that supplement intake and ethnic background were associated with the biggest differences. Skin color was not a major factor, suggesting that genetic factors are responsible for the observed differences between participants of different ethnic backgrounds.

## Introduction

Serum 25-hydroxyvitamin D (25OHD) levels currently represent the best biomarker for assessing vitamin D nutriture because they reflect ingested vitamin D as well as that derived from cutaneous synthesis [1]. The adequacy of a population can be assessed by calculating the percentage of participants with 25OHD values falling below the US Institute of Medicine (IOM) cut point values of 30 nmol/L (risk of deficiency relative to bone health) [1] and 40 nmol/L (estimated average requirement for a population) [1, 2]. The IOM also reported that at serum 25OHD ≥ 50 nmol/L, 97.5% of the population would be sufficient, corresponding to a recommended dietary allowance (RDA) of 600 IU/d for individuals ≤ 70 years of age and 800 IU/d for those above 70 years of age. These reference intake values assume minimal sun exposure.

A recent national level survey of Canadians indicated that overall rates of deficiency and insufficiency were modest (7.4% and 19.4%, respectively) [3]. However, an ethnicity related disparity was apparent so that a higher percentage of participants who were not of European ancestry had 25OHD values <30 nmol/L (16.3% vs. 3.0% for Europeans) and <40 nmol/L (30.5% vs. 8.7%) [4]. Interpretation of factors influencing 25OHD levels in individual ethnic groups was not possible because the non-European group included 11 different ethnic/cultural groups [4]. It is important to study individual groups given the many genetic factors that associate with vitamin D status [5, 6], including variations in skin color and reported vitamin D binding protein [7, 8] as well as dietary and sun exposure preferences. South Asian Canadians, which include people of Indian, Pakistani, Sri Lankan, Bangladeshi, and Nepali ancestry make up a large and rapidly growing segment of the Canadian population (4.8% in 2011[9]). It is possible that a combination of darker skin, genetic factors, and life style contribute to lower vitamin D status in ethnic South Asians living in countries away from the equator [10]. Higher risk of femoral neck osteoporosis among post-menopausal females of South Asian ancestry has been noted in the United States [11], underscoring the need for close monitoring. Comprehensive data are lacking for the Canadian population, except for a small study of South Asian and East Asian students from the University of Toronto [12] and a study of Non-Western immigrant children between the ages of 1–6 living in Toronto [13].

The primary objective of this study was to determine the vitamin D intake and serum 25OHD levels of healthy adult volunteers aged 20 to 79 in Canadians of self-identified South Asian and European ancestry living in the same geographical area during two seasons (spring and fall). An important outcome was the prevalence of individuals falling below the IOM cut points of 30 and 40 nmol/L. This study is a component of a broader study whose objectives included an assessment of dietary and metabolic cardiovascular risk factors. The current manuscript reports only on physical characteristics, vitamin D intake (diet plus supplements) and blood status in adults in order to determine their interrelationship. The results for omega-3 status [14] and cardio-metabolic disease risks [15] have already been published.

## Materials and methods

### Study design, recruitment and population

This study was conducted according to the guidelines laid down in the Declaration of Helsinki. The study protocol was approved by Health Canada and the Public Health Agency of Canada Research Ethics Board (REB 2010–0043). All participants provided written informed consent. This was part of a larger study that included cardiovascular health outcomes [14]. Measures were taken at the end of winter (April 14^th^ to May 6^th^, 2012; spring) and at the end of summer (September 12^th^ to October 13^th^, 2012; Fall).

It was estimated that 40 participants per sex (2 levels), age (3 levels) and ethnic background (2 levels) categories (= 480 total participants) were required to detect a difference of about 23 nmol/L (standard deviation for each group of 20 nmol/L) [12] with a statistical power of 80% at a significance level of 0.05 (PROC GLMPOWER; SAS Enterprise Guide v5.1; SAS Institute, Cary NC). The value of 480 represented a minimum number since the difference noted above was between ethnic backgrounds—sex/age differences were likely smaller [12]. Participant eligibility for the study was assessed using a questionnaire that was completed prior to study enrollment. The inclusion criteria were: age (20 to 79 years), self-identified South Asian or European ancestry (assessed by questionnaire), and resident of the National Capital Region of Canada (around the city of Ottawa Ontario, Canada; approximate latitude of 45.4°N). This area was the chosen target area because of available resources and the desire to avoid potential differences in sun exposure over the course of the year related to location. Medication use and consumption of vitamin D supplements were not used as exclusion criteria because we were interested in evaluating the status of free-living participants. A total of 669 participants were enrolled in the study of which 561 contributed data for both spring and fall. The remaining participants contributed data for either spring or fall only. No participants self-identified as illiterate.

Nine different data collection sessions were held in the morning at locations around the Ottawa area in the spring (6 different locations) and fall (7 different locations). The locations were chosen for participant convenience. Participants were encouraged to sign up prior to attending a session but were accommodated at the location if they opted for a different session. Fasting blood samples were taken by one of two registered nurses (phlebotomists) and laboratory technicians measured height, weight and skin color. Body mass index (BMI) was calculated from measured height and weight. After data collection, a selection of snacks and drinks was provided. The principal investigator was present at all clinics to answer questions from participants. Bilingual dietitians were present to help with interpretation should it prove necessary.

### Dietary questionnaire

Each participant received a self-administered questionnaire [14] by e-mail or by regular post mail (choice of French or English) 2 weeks before attending a data collection session where the completed questionnaires were collected. If the questionnaire had not been filled out, participants were required to complete the form on-site prior to physical data collection. At least two bilingual dietitians were present to assist with this task should it prove necessary. The food frequency portion of the questionnaire (FFQ) was used to quantify omega fatty acid (previously reported data) and vitamin D intake (this study) during the previous 4 weeks. Food items in the FFQ were chosen using data from five main sources: a food frequency questionnaire developed to target vitamin D intake in young Canadian adults of diverse ancestry [16], commonly consumed food items identified in the Canadian Community Health Survey cycle 2.2-Nutrition [17], a FFQ designed to target EPA and DHA intake [18], foods that could represent significant sources of vitamin D or omega fatty acids (e.g., chicken, beef and pork liver), and the Canadian Health Measures Survey [19] fish intake questionnaire. Smelt and Kingfish/seer fish were also included because they were believed to be frequently consumed by participants of South Asian ancestry in the survey area. Because the list of fish was long and potentially burdensome to participants, it was simplified to reflect types of fish most likely to be consumed based on data from the Canadian Community Health Survey and data gathered from 2 pilot studies. For example, the original list of four types of canned tuna was collapsed to a single item. An average nutrient value was then created for that item.

Vitamin D intakes were estimated from the FFQ using a standard reference food composition database (the Canadian Nutrient File version 2015 [20]), food company websites, and food labels. The survey also included questions on supplement use. Participants were instructed to provide the Natural Product Number (NPN) and package/nutrition label of the supplement and the frequency of consumption, which were then used to estimate supplement-associated vitamin D intakes.

### Serum total 25OHD analysis

Fasting blood samples were collected by venipuncture into 2 mL serum separator tubes. The serum fraction was isolated after centrifuging (30 min x 2280g) and stored at -80°C until analysis. Serum 25OHD concentrations were measured using the DiaSorin LIAISON TOTAL 25OHD chemiluminesence assay using a LIAISON autoimmunoanalyzer and LIAISON TOTAL 25OHD Integrals (Diasorin Inc, Stillwater, MN, USA). The assay was performed according to the manufacturer’s instructions. Assay performance was assessed using quality control materials purchased from Biorad Diagnostics Inc. (Montreal, Quebec, Canada). The inter- and intra-assay variability ranged from 6.4–9.4% and 4.9–6.1% respectively. The laboratory has been in proficient standing with the Vitamin D External Quality Assessment Scheme since 2007, and also participates successfully in the College of American Pathologists accuracy based surveys. Values were corrected to standardized values [3].

### Skin color measurement

Three skin color measurements were taken (underarm of the biceps) using a portable Minolta Smart Probe 400 colorimeter (IMS INC., Millford CT). The value measured at the spring data collection time point was used as the natural skin color (no sun exposure) unless only fall data was available. The Smart Probe 400 translates absorbance into the International Commission on Illumination (CIE) L*a*b* color scale. The L and *b values were transformed into Individual Topology Angle values (ITA) using previously published equations [21]. ITA values show a good correlation with melanin quantity and distribution [21].

### Statistical analysis

Only individuals with serum 25OHD values <150 nmol/L and total vitamin D intakes <4000 IU/d were included. This was done to remove the effects of individuals consuming in excess of the safe upper limit defined by the IOM [1] and potential recent visits to a southerly location. Participants with high 25OHD values may be at higher risk for overall morbidity [22], although this relationship has recently been re-evaluated [23, 24]. Statistical analysis was performed using a Type 1 generalized mixed model (PROC MIXED; SAS Enterprise Guide v5.1; SAS Institute, Cary NC) with unstructured covariance between seasons for each ethnic group. This allowed for the inclusion of all participants regardless of their participation record and the calculation of the variance components for each ethnic background within each clinic visit along with the between season covariances [25]. These variances and covariances were used in the testing of the effects as appropriate.

A forward selection method was used for model building with model(s) fitted using a maximum likelihood method. This involved building two models, one including only main effects and the other with main effects and all two-way interactions. The difference between the two log maximum likelihood values was compared to a chi-square distribution (degrees of freedom = difference between the number of parameters of the two models) to test the significance of the two-way interactions. P-values were then calculated for each two-way interaction and those with low p-values were kept in the model. The model was then rerun with the selected interaction term included. The differences between the two log maximum likelihood values (all two-way versus reduced two-way) were compared to a chi-square value as above, and this process iteratively repeated until the comparison was not significant. This was then repeated for the three-way interactions with the provision that the effects tested in a three-way interaction must also include all two-way interactions that incorporate the variables even if they had been initially rejected. To avoid over fitting the model, all higher order interactions were excluded from the analysis. Only two three-way interactions were statistically significant, thus all two-way interactions that shared variables with them were retained in the model. Adjusted least squares estimates (LSE, adjusted for the other factors in the model) were compared using the Tukey-Kramer correction for multiple comparisons.

Differences in participant characteristics (S1 Table) were tested using a 2 way ANOVA with interaction (PROC GLM, SAS Enterprise Guide v5.1). Comparisons were adjusted using the Tukey-Kramer correction for multiple comparisons. The Fisher Exact test was used to assess whether the proportion of participants below selected cut points was the same for both seasons. The effect of season on 25OHD values was tested using a paired t-test. These tests differed from the mixed model approach because they included only participants that attended both clinics (N = 561). Differences were considered significant when P <0.05.

## Results

### Demographic information

Full details of the demographic information are documented in a previous publication [14]. Of the 669 participants, 49% were of South Asian ancestry. A higher percentage of participants of European ancestry (68.0%) were female when compared to those of South Asian ancestry (46.6%; S1 Table). On average, participants of South Asian ancestry (vs. European ancestry) and females (vs. males) weighed less and were shorter. Males (vs. females) of European ancestry had higher BMI values but no difference was observed among participants of South Asian ancestry.

### Serum 25OHD concentrations

Serum 25OHD concentrations were approximately 23 nmol/L higher in European participants compared to South Asian participants (70.8 nmol/L (68.1, 73.5) vs. 42.7 nmol/L (40.5, 45.0); P<0.001). Females had 11 nmol/L higher values when compared to males (66.1 nmol/L (64.3, 68.0) vs. 54.8 nmol/L (52.9, 56.7); P<0.001). Overall, 25OHD values were highest in the 60–79 year olds (67.9 nmol/L (64.8, 70.9), followed by the 40–59 year olds (60.1 nmol/L (58.1, 62.1)) and the 20–39 year olds (58.8 nmol/L (56.5, 61.1)). There was a greater difference between young (ages 20–39) and old (ages 60–79) in South Asian participants vs. European participants (14.5 nmol/L vs. 6.1 nmol/L; Table 1). Differences between seasons were significant only for those of European ancestry (2.9 nmol/L (1.0, 4.9) higher in fall; P = 0.01) while no difference was noted for those of South Asian ancestry (0.01 nmol/L (-1.3, 1.3); NS).

**Table 1:** Serum 25OHD concentrations in participants of South Asian and European descent by sex and age, both seasons combined^1^.

Sex	Age	South Asian ancestry		European ancestry	
		N	nmol/L	N	nmol/L
Both	20–39	174	42.7 (40.5, 45.0)^a^	233	70.8 (68.1, 73.5)*^a^
Both	40–59	287	49.5 (47.1, 51.8)^b^	272	71.3 (68.7, 74.0)*^a^
Both	60–79	125	57.2 (53.6, 60.9)^c^	147	76.9 (72.7, 81.1)*^a^
Both	20–79	586	49.1 (47.5, 50.7)	652	72.4 (70.7, 74.1)*
Females	20–39	91	45.4 (41.9, 48.8)^a^	167	72.0 (68.7, 75.3)*^a^
Females	40–59	138	54.0 (50.4, 57.6)^a^	188	73.0 (69.8, 76.1)*^a^
Females	60–79	44	65.1 (59.3, 70.9)^b^	88	81.6 (75.5, 87.6)*^b^
Females	20–79	273	52.9 (50.5, 55.3)	443	74.3 (72.1, 76.5)*
Males	20–39	83	39.8 (37.0, 42.6) ^a^	66	67.8 (63.1, 72.5)*^a^
Males	40–59	149	45.3 (42.3, 48.2)†^b^	84	67.7 (62.9, 72.4)†*^a^
Males	60–79	81	53.0 (48.5, 57.5)^c^	59	69.9 (64.8, 75.1)†*^a^
Males	20–79	313	45.8 (43.8, 47.8)†	209	68.4 (65.6, 71.1)†

^1^Values represent mean (CI) for the indicated number of observations (N).

ANOVA showed an effect of ethnic background, Age and Sex as well as an age*ethnic background interaction.

Superscripts show significantly difference from South Asians (*) age and females (†) as determined by ANOVA.

Ages with different letters are significantly different at the P<0.05 level as determined by Tukey’s studentized range test.

### Proportion of individuals below IOM cut points and below 50 nmol/L

In general, a higher prevalence of South Asian participants and males fell below cut points (Table 2) but the differences were dependent on sex and season. For example, in the spring, a similar percentage of South Asian and European females fell below the 30 nmol/L cut point but significantly more South Asian males fell below this value. Among participants of South Asian descent, a significantly higher percentage of males fell below cut points vs. females with the exception of the 30 nmol/L cut point in the fall season. However, among ethnic Europeans the percentage of males and females below cut points was similar with the exception of the fall 50 nmol/L cut point.

**Table 2:** Percentages of participants of South Asian and European ancestry with 25OHD values falling below IOM cut points and below 50 nmol/L by sex and season^1^.

		South Asian ancestry			European ancestry		
Gender	Season	<30 nmol/L	<40 nmol/L	<50 nmol/L	<30 nmol/L	<40 nmol/L	<50 nmol/L
Female	Spring	7.1	29.1*	51.1*	3.5	9.1	19.8
Male	Spring	20.6*‡	44.2*‡	67.3*‡	2.8	10.4	18.9
Female	Fall	12.1*	27.3*	51.5*	0.0†	1.4†	8.1†
Male	Fall	14.9*†	36.5†‡	68.9‡	1.0	3.9†	13.6‡

^1^Values represent the percentage of participants with 25OHD concentrations falling below indicated cut points.

Significantly different from the corresponding value for

*participants of European ancestry

†spring; and

‡females as determined by Fisher’s exact test.

### Factors related to serum 25OHD concentrations

A mixed regression model was used to investigate factors related to 25OHD concentrations. It included nine factors tested in the following order: Log(vitamin D intake from food), a variable indicating whether the individual consumed vitamin D-containing supplements, BMI, season, age group (20-39y, 40-59y and 60-79y), sex, skin color (linearized by ITA), total serum cholesterol, and ethnic background. Two- and three-way interactions between these parameters were also included when they were significant. Ethnic background was placed last so that its effect could be assessed after taking the other factors into account.

Ethnic background was the major categorical factor influencing serum 25OHD levels within this study, followed by taking supplements and sex (Table 3). The difference in serum 25OHD between ethnic backgrounds after adjustment (26 nmol/L) was similar to the unadjusted difference (Table 1). This suggests that this difference was due to inherent differences between the two ethnic groups and not to any confounding factor. The adjusted difference between females and males (5.0 nmol/L higher values for females) was lower than the 11.3 nmol/L difference observed in Table 1 and may reflect differences in supplement intake between the sexes. Relatively minor seasonal differences were not significant.

**Table 3:** Magnitude of factors related to serum 25OHD values derived by regression^1^.

Factor (Unit)	Comparison/Group	value	P
**Categorical Predictors (difference of LSE):**			
Ethnic background	SA vs. EU	-26.1 ± 2.5	<0.001
	SA vs. EU: Fall	-27.9 ± 1.8	0.001
	SA vs. EU: Spring	-24.3 ± 2.6	<0.001
Takes supplement	SA: N vs. Y	-10.8 ± 1.3	<0.001
	EU: N vs. Y	-10.7 ± 1.6	<0.001
Sex	Female vs. Male	5.0 ± 1.4	<0.001
Season	SA: Fall vs. Spring	-1.4 ± 1.1	NS
	EU: Fall vs. Spring	2.3 ± 2.0	NS
**Continuous Predictors (slope for factor by group):**			
BMI	20–39 y age group	-0.65 ± 0.59	
nmol/L·(kg/m^2^)^-1^	40–59 y age group	-1.05 ± 0.58	
	60–79 y age group	-1.93 ± 0.36	
Skin Color	SA	0.10 ± 0.19	
nmol/L·ITA units^-1^	EU	-0.41 ± 0.13	
	Fall	-0.27 ± 0.17	
	Spring	-0.41 ± 0.13	

^1^Values adjusted by a model that included the following factors: vitamin D intake from food, supplement consumption (Y/N), BMI, season, age group, sex, skin color, total serum cholesterol, and ethnic background.

SA (South Asian ancestry), EU (European ancestry).

**Fig 1:**
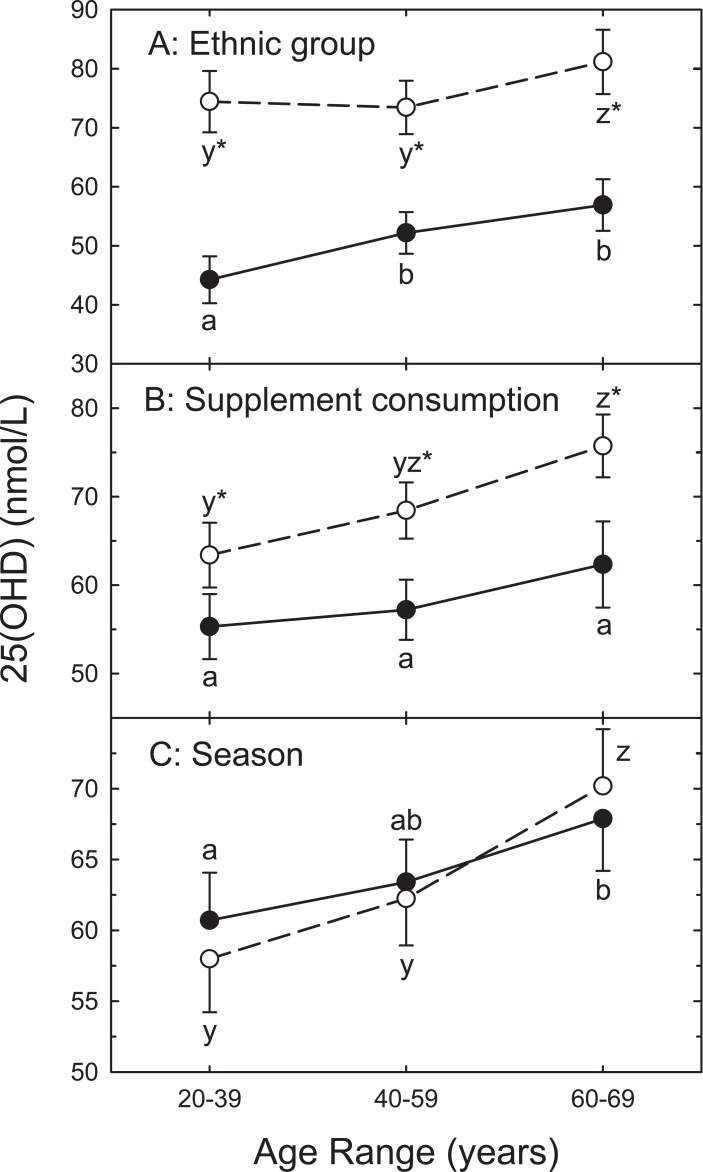
**LSE serum 25OHD values with 95% confidence intervals as a function of ethnic background (A), vitamin D supplement consumption (B), and season (C) by age group.** Panel A: values are for Europeans (open) or South Asians (filled). Panel B: those not consuming supplements (open) or supplement consumers (filled). Panel C: spring (open) or fall (filled). Superscripts (a,b,c,y,z) denote significant differences (P<0.05) when compared to other age ranges within South Asian (y, z) or European (a, b, c) ethnic groups. *Different from value for corresponding ethnic group (panel A), supplement status (panel B) or season (panel C; P<0.05).

Serum 25OHD values tended to increase with age but this depended on ethnic background, supplement consumption and season (Fig 1A). For example significantly higher 25OHD values were observed in the oldest group among European participants. However, both the middle and oldest groups were higher among South Asian participants. Taking vitamin D supplements was associated with higher serum 25OHD at all ages but no age-related differences were observed for those not consuming supplements (Fig 1B). No seasonal differences were observed for individual age groups (Fig 1C). There was a significant correlation between 25OHD values measured in spring and fall (S1 Fig; partial correlation coefficient, adjusting for ethnic background was 0.77; P < 0.001) suggesting similar behavioral and dietary patterns existed between seasons [12].

**Fig 2:**
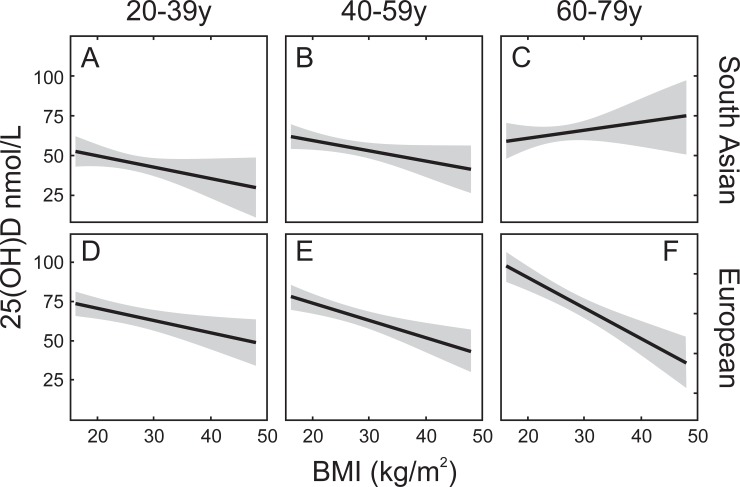
Relationship between serum 25OHD and BMI as a function of age and ethnic background. Solid line represents the relationship obtained from the regression model with 95% confidence limits (shaded area). Panels A (N = 173, age range 20-39y), B (N = 284, age range 40-59y) and C (N = 125, age range 60-79y) represent data from participants of South Asian descent. Panels D (N = 233, age range 20-39y), E (N = 271, age range 40-59y), and F (N = 147, age range 60-79y) represent data from participants of European descent.

Participants with lower BMI had higher 25OHD for all groups except for the older participants of South Asian ancestry (60–79 year old; Fig 2C). Unlike the other age/ethnic background groups, there appeared to be a positive correlation between intake of vitamin D-containing supplements and BMI in this group (S2 Fig).

### The relationship between vitamin D intake and serum 25OHD concentration

Vitamin D intake from food (P <0.001) correlated positively with serum 25OHD levels but the difference in intake (189 ± 5 IU/d for European participants (n = 643) vs. 176 ± 5 IU/d for South Asian participants (n = 541); NS) was too small to account for the ethnic background-associated difference in 25OHD levels. There was no seasonal effect on vitamin D intake from food (178 ± 5 IU/d in the fall vs. 188 ± 5 IU/d in the spring; NS). The low intake levels were probably due to low milk consumption since, in Canada, milk can be a major contributor to food vitamin D (400 IU vitamin D/serving). The median milk intakes were much lower than the 2 servings/d (500 mL) recommended by Health Canada [26]: 0.37 servings/d (South Asian participants) and 0.27 servings/d (European participants).

Consumption of vitamin D-containing supplements also correlated with serum 25OHD levels (P<0.001). Almost 59% of the participants reported consuming supplements, accounting for an average of 75% of the total intake in consumers. More participants of European (63%) than South Asian (49%) ancestry reported consuming supplements and there was a difference in the amounts reported: European participants reported taking a median value of 714 (342–1307; quartile 1–3) IU/d while South Asian participants reported consuming 654 (357–1000) IU/d (P<0.001, Mann-Whitney U test). Median values are reported because of the highly variable consumption pattern. Higher supplement consumption resulted in a higher total (food + supplement) intake for those of European ancestry (484 IU/d; 198–1146 IU/d) compared to those of South Asian ancestry (338 IU/d; 155–878 IU/d; P<0.001, Mann-Whitney U test). No seasonal differences in supplement intake were noted but intake increased with age: 52% of respondents aged 20-39y took supplements vs. 60% for the 40–59 year olds (NS vs 20-39y) and 74% for the 60–79 year olds (P<0.001 vs 20-39y; P = 0.004 vs 40-59y; z test).

**Fig 3:**
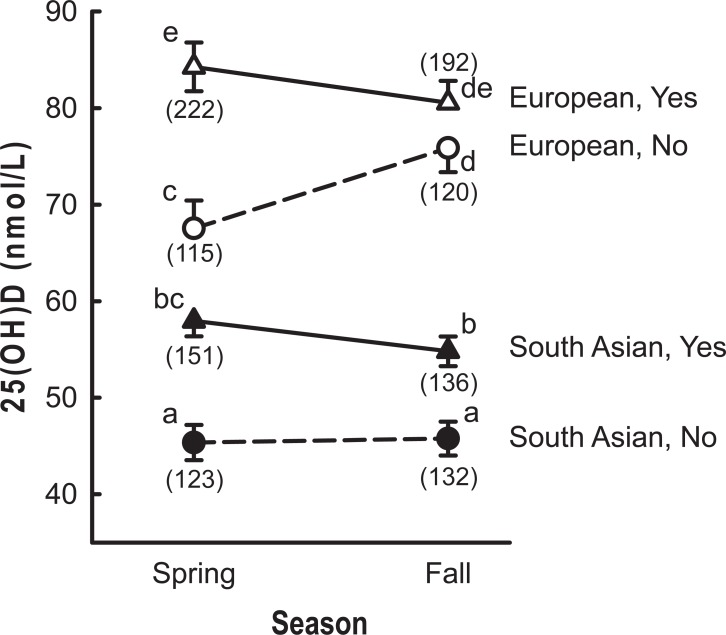
LSE serum 25OHD values as a function of season, ethnic background and consumption of vitamin D containing supplements (Yes/No). Values represent mean ± standard error. Open symbols represent participants of European descent and filled symbols represent those of South Asian descent. Triangles represent participants consuming supplements while circles represent participants who did not consume supplement. Symbols with different superscripts differ significantly (P<0.05). The number of participants in each category is indicated below the symbol in parentheses.

The relationship between supplement intake and serum 25OHD levels was dependent on ethnic background and season (Fig 3; P = 0.001). For European participants, seasonal differences were noted only in those who did not take supplements with significantly higher values observed in the fall. On the other hand, no seasonal differences were noted in South Asian participants. Comparing participants of European and South Asian descent in the spring (no effect of sunlight) showed a difference of 20 nmol/L for both supplement users and non-supplement users. This value is similar to the difference of 28 nmol/L noted in Table 1 (unadjusted values) suggesting that differences in supplement intake cannot account for the ethnic background-related difference in 25OHD concentrations.

### Skin color

**Fig 4:**
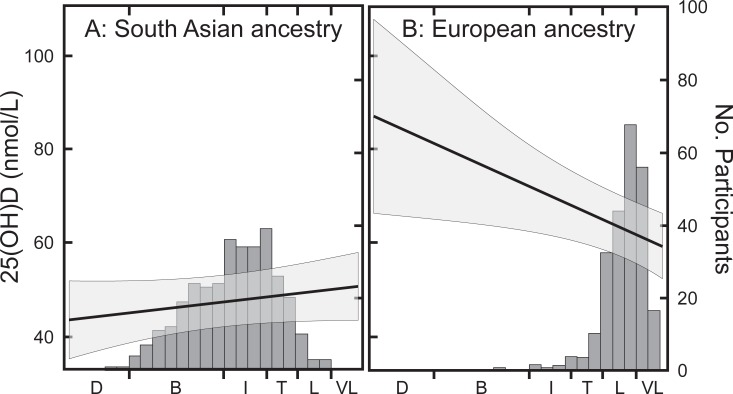
**Serum 25OHD values as a function of skin color for participants of South Asian (Panel A) and European (Panel B) ancestry.** The x axis shows skin color classifications corresponding to Individual topology Angles calculated for each individual [21]: D (dark), B (brown), I (intermediate), T (tanned), L (light), and VL (very light). The straight line represents the least squares regression relationship between serum 25OHD values and skin color obtained from the PROC MIXED model (left axis). The shaded area represents the 95% confidence limits. The bars represent the number of participants falling within the indicated skin color range (right axis).

Skin color was closely related to ethnic background as shown by the distribution of participants among the different classifications (filled bars, Fig 4). In particular, skin color among South Asian participants was fairly evenly distributed across the range from “light” to “brown” (97% of participants) while the range was more narrow in European participants (“very light” to “tanned”). A relationship between 25OHD values and skin color was noted, but the direction depended on ethnic background. In participants of South Asian ancestry, 25OHD values were lower at darker skin color while the opposite was observed in ethnic Europeans (ethnic background × skin color interaction; P<0.001). The increase seen among participants of European ethnicity was probably due to tanning behavior in a few individuals, which would have greatly influenced the slope at values > “Intermediate” (note the high variance). Thus, the impact of skin color on 25OHD concentrations was estimated using only the data from South Asian participants because this group had a wide range of skin colors and because using a single ethnic background excluded other ethnic background-related factors. A 5.6 nmol/L difference in 25OHD between the lightest and darkest skin colors (97% of participants) showed a relatively small contribution of skin color. The contribution of skin color was also estimated by comparing 25OHD concentrations in participants of European and South Asian descent within the “light” and “tanned” classifications. Similar to the full population analysis, those of South Asian ancestry from this smaller grouping had 25OHD values that were 23 nmol/L lower than those of European descent suggesting that genetic differences arising from ancestry were much more important than the effect of skin color.

## Discussion

An important finding of this study is that participants of South Asians ancestry were more likely than participants of European ancestry to fall short of the IOM recommended 25OHD levels for the prevention of deficiency and inadequacy that are based on bone health. 29–44% of South Asian participants had serum 25OHD <40 nmol/L while ≤10% of European participants fell below this cut point. These results are consistent with those of the Canadian Health Measures Survey (CHMS; Cycle 1, 2007–2009) where non-European Canadians had an overall inadequacy prevalence of 30.5% compared to 8.7% for European Canadians [4]. These data are also in line with differences reported between individuals of European and Asian ancestry in Toronto during the fall: participants of European ancestry had had 25OHD concentrations of 75 nmol/L compared to 50 nmol/L for those of East and South Asian ancestry [12]. This range of difference has been reported in several different western countries.

The relatively high rate of risk of deficiency among those with South Asian ancestry (7–21%) is of concern because, according to the IOM [1], this group is at an increased risk of osteomalacia. Our findings are consistent with CHMS survey results that showed 3% of European Canadians and 16.3% of non-European Canadians were at risk of deficiency [4], although in the CHMS study the non-European CHMS category included persons of many ethnic origins [4]–only approximately 25% were of South Asian ancestry. Our findings are also consistent with previous work in Canada showing a high prevalence of low vitamin D levels among young adults of South Asian ancestry living in Southern Ontario [12]. In that study, 35.5% of 18–30 year old South Asian participants had 25OHD concentrations <25 nmol/L. The high prevalence of vitamin D levels < 30 nmol/L in younger adults is important, given its role (along with calcium) in bone accretion, even in young adults [27, 28] and there is evidence that increased bone mineral density at younger ages can prevent later fractures at some locations [29].

The present study also confirmed that vitamin D-containing supplements represent an important contributor to final serum 25OHD levels in Canada [4]: supplement consumers had approximately 11 nmol/L higher values regardless of ethnic background. The impact of supplements on 25OHD levels in this study was not surprising considering that daily consumption of the minimum level of vitamin D supplement (400 IU) would have increased reported intakes (around 180 IU/d) three-fold and consumption of the highest reported supplement (2000 IU/d) would have increased intakes 11 fold. The impact of supplements on status was shown by comparing the percentages of participants with deficient and insufficient levels: 1.3% of those taking supplements vs. 6.3% of those not taking supplements had 25OHD concentrations < 30 nmol/L and 4% of those taking supplements vs. 16% of those not taking supplements had serum values < 40 nmol/L. The impact of supplements was also shown by similar 25OHD values in European non-consumers and South Asian consumers in the spring.

The present study confirmed that total vitamin D intake (from food and supplement combined) is an important factor related to final serum 25OHD levels. However, it was not possible to determine the magnitude of the effect of food intake alone on 25OHD values from the self-reported food data of the present study. Several factors contributed to this issue. Firstly, large national food composition databases include vitamin D levels in foods whenever possible but they do not presently include the 25OHD content of animal-based foods, which has been reported to have a greater effect on serum 25OHD levels than vitamin D [30, 31]. Including meat-associated 25OHD would have increased vitamin D-equivalent intake estimates. Secondly, although a food frequency questionnaire (FFQ) is useful for assessing past food intake, it has limitations. Compared to other tools, the FFQ is the least expensive and has the lowest response burden but a common disadvantage is long-term recall error [32] because the FFQ requires participants to remember consumed foods over a defined period of time. Thus, compared to other dietary assessment tools like dietary recalls, FFQs may have greater measurement error [32]. Thirdly, an analysis of red blood cell fatty acid profile data from our earlier study suggested a different fish intake pattern than that reported on the FFQ by the participants [14]. Since fish are an important source of vitamin D, this could have overestimated intake estimates. These shortcomings mean that it was not possible to use our data to determine the amount of vitamin D from food sources needed to maintain adequacy in either summer or winter.

Despite these limitations, it is apparent that the Canadian food supply contains sufficient vitamin D to support adequate 25OHD levels during the winter months since approximately 60% of those not taking supplements (both ethnic backgrounds combined) at the spring sampling point had 25OHD values in excess of 40 nmol/L. At this time point, sunlight effects are expected to be negligible. This suggests that appropriate dietary choices can provide sufficient vitamin D but it is also apparent that many individuals achieve sufficiency only by consuming supplements (approximately 60% of participants took supplements). This is illustrated when one considers that 93% of participants taking supplements had 25OHD levels >40 nmol/L. Any potential differences in supplement intake were not the reason for the discrepancy between the two ethnic groups in our study. This was shown by comparing European and South Asian participants who did not consume vitamin D-containing supplements: South Asian non-consumers were 16 nmol/L lower than European non-consumers when measured in the spring.

Endogenous synthesis of pre-vitamin D in response to sunlight during the summer is an important source of vitamin D that is especially relevant in a region with a relatively short period of productive sunlight like Ottawa [33]. Two different patterns were observed in response to season in our study. European participants who did not consume supplements had approximately 10 nmol/L higher values at the end of the summer while European participants who consumed supplements showed no change. This suggests that summer sun exposure is important when 25OHD values are lower (no supplement consumption) but sunlight has a less pronounced effect when 25OHD values are relatively high. However, this pattern was not observed in South Asian participants: a lack of a summer-associated response in both supplement consumers and non-consumers suggested that ethnic background-related factors were more important in determining 25OHD levels in this group. Previous work has shown that vitamin D synthesis is lower in darker skinned individuals for equivalent light exposure [34], which is thought to be the reason for lower serum 25OHD values in darker skinned individuals living in northern countries [35, 36]. On the other hand, calculations of vitamin D synthesis at latitudes equivalent to Boston during July show that only 6 min longer is needed for darker-skinned individuals to synthesize 400 IU of vitamin D/d [37]–a time that should be functionally insignificant. So how important a factor is skin color? In a comparison of factors influencing final 25OHD levels, data has shown that vitamin D intakes were more important than sunlight synthesis in determining final 25OHD values [38], a conclusion supported by the present study. In addition, skin pigmentation only marginally affected South Asian 25OHD values in our study after correcting for ethnic background, sex, age and vitamin D intakes, even though there was a wide variation in skin color within this group (Fig 4A). This agrees with a recent New Zealand study, where skin color explained relatively little variation among ethnic groups after adjusting for ethnic background [39]. It is also in agreement with a study examining the relationship between skin color and 25OHD in Pacific People and people of European ethnicity [40]. This study concluded exposed skin color was more important that unexposed color in determining 25OHD levels. We had also measured color on the back of the hand (exposed skin) in our study but this value was not related to 25OHD concentration and was excluded from the analysis. We also looked at the difference in color between exposed and upper arm (unexposed) skin to estimate sun exposure but this value was not significantly related to serum 25OHD and was also excluded from the final model. Reports have demonstrated that synthesis is dependent on an adequate area of bare skin exposed to ultraviolet B radiation and on time spent outside during the most productive period of the day. Our study did not collect information on the amount of time spent outdoors or on the time of day spent outside during the summer, an important limitation. The influence of these factors, therefore, remains to be more thoroughly investigated. At present we can only conclude that our data suggests that the lower vitamin D status of South Asian participants was not due to differences in skin color *per se*. It is possible that other factors, not related to measured pigmentation, could have influenced the final production of 25OHD during exposure to full sunlight. Evidence for a limiting effect of full spectrum solar radiation on pre-vitamin D synthesis has been published [41] due to photoisomerization of pre-vitamin D in skin. Differential absorption of wavelengths associated with this process would be expected to influence final cutaneous pre-vitamin D levels.

The individual model parameters were used to estimate the contributions of sex, supplement consumption, age, BMI, cholesterol levels, vitamin D intake from food and skin color to the difference between the ethnic groups. To do this, we calculated differences in 25OHD attributable for each factor using group averages. For example, the overall effect of BMI on 25OHD levels, obtained from the full model, was used to calculate expected 25OHD levels using the average BMI of each group. The difference between these two calculated levels is the expected difference due to BMI. Combining these factors showed that about 85% of the ethnic difference (spring or fall) could not be accounted for from the factors included in our model. This suggested that unidentified genetic factors must be responsible for the observed difference. One potential factor was differences in the vitamin D binding protein (VBP) levels and isoforms. While initial research suggested differences in the VBP concentrations among ethnic groups [8], more recent measurements using newer methods indicate that this may have been artefactual [42, 43]. In addition, there is controversy surrounding the derivation of ethnic background-related VBP affinity constants [44]: one study using vitamin D as the probe reported differences [45] while three studies using labelled 25OHD showed no difference [46–48]. This suggests that VBP is not an important factor related to ethnic differences in serum 25OHD levels [49].A strength of our study is its focus on the vitamin D status of Canadians of South Asian ancestry within Canada’s National Capital Region. It has been estimated that this community accounts for 2.1% of the South Asian population in Canada [9] and these data may reflect trends within the Canadian South Asian population. On the other hand, a weakness of the study is the use of volunteers rather than demographically targeted participants. Volunteers likely have higher education and socio economic status than the general population and this may account for the high percentage of vitamin D supplement users reported. This study focused primarily on collecting food and supplement intake data as well as anthropometric data while other factors affecting blood vitamin D levels, including length of daily sun exposure, sun avoidance measures, and genotype markers were not collected. Also, while the current study was focused on the South Asian community, it would be of interest to expand this analysis to determine whether the major factors identified in this study apply equally to other ethnic groups.

In summary, we show that vitamin D levels below 40 nmol/L are common in Canadians of South Asian ancestry residing in the National Capital Region, especially among the young-adults in the 20–39 age group. There was a tendency for fewer of the South Asian participants in our study to consume vitamin D-containing supplements and this, coupled with a lack of response to summer-related vitamin D synthesis were important factors related to lower 25OHD levels when compared to people of European descent living in the same area. However, these factors could not account for the observed difference between these two groups. The relationship between supplement intake, season and ethnic background is complex and it appears that factors other than skin color and supplement intake are important.

## Supporting information

S1 Table Characteristics of the survey participants at their initial visit. (DOCX)

S1 Fig Relationship between serum 25(OH)D values measured in spring and fall for European (open circles) and South Asian (filled circles) participants. Data includes only participants who attended both spring and fall collections. The relationship between spring and fall values was assessed by partial Pearson correlation analysis with race as the other variable. The 95% prediction ellipse is shown.
(PDF)

S2 Fig Relationship between serum 25(OH)D concentrations and BMI (kg/m^2^) for participants of European and South Asian ancestry for indicated age ranges. Solid line represents regression of least square means obtained from the PROC MIXED model with shaded area representing the 95% confidence limits.
(PDF)
