# Supplementary material for: Low 25-hydroxyvitamin D levels are more prevalent in Canadians of South Asian than European ancestry inhabiting the National Capital Region of Canada
Source: PLoS One. 2018 Dec 12;13(12):e0207429. doi: 10.1371/journal.pone.0207429 (PMC6291105; doi:10.1371/journal.pone.0207429)
Supplement: S1 Table — (DOCX) [file pone.0207429.s001.docx]

**S1 Table**: Characteristics of the survey participants at their initial visit^1^.

|  |  | All participants | South Asians | Europeans |
| --- | --- | --- | --- | --- |
| Both genders | N^2^ | 669 | 325 | 344 |
|  | Age (years) | 47.8 ± 13.6  (46.7, 48.8) | 47.6 ± 13.7  (46.1, 49.1) | 47.9 ± 13.6  (46.4, 49.3) |
|  | % Females | 57.5 | 46.6 | 68.0^a^ |
|  | Weight (kg) | 72.1 ± 15.1  (71.0, 73.3) | 70.3 ± 13.6  (68.8, 71.8) | 73.9 ± 16.2^a^  (72.2, 75.6) |
|  | Height (cm) | 166 ± 9  (165, 167) | 164 ± 9  (163, 165) | 168 ± 9^a^  (167, 169) |
|  | BMI | 26.0 ± 4.6  (25.7, 26.4) | 25.9 ± 3.8  (25.5, 26.3) | 26.2 ± 5.3  (25.6, 26.7) |
| Females | N^2^ | 387 | 152 | 235 |
|  | Age (years) | 46.8 ± 13.3  (45.5, 48.1) | 46.0 ± 13.1  (43.9, 48.0) | 47.3 ± 13.4  (45.6, 49.1) |
|  | Weight (kg) | 67.2 ± 14.5  (65.8, 68.7) | 64.0 ± 11.9  (62.1, 65.9) | 69.3 ± 15.6^a^  (67.3, 71.3) |
|  | Height (cm) | 162 ± 7  (161, 162) | 157 ± 6  (156, 158) | 164 ± 7^a^  (163, 165) |
|  | BMI | 25.7 ± 5.1  (25.2, 26.2) | 25.7 ± 4.0  (25.1, 26.4) | 25.7 ± 5.7  (25.0, 26.4) |
| Males | N^2^ | 282 | 173 | 109 |
|  | Age (years) | 49.1 ± 14.0^b^  (47.4, 50.7) | 49.1 ± 14.1^b^  (47.0, 51.2) | 49.1 ± 14.0  (46.4, 51.7) |
|  | Weight (kg) | 78.9 ± 13.2^b^  (77.3, 80.4) | 75.9 ± 12.6^b^  (74.0, 77.7) | 83.7 ± 12.6^ab^  (81.3, 86.1) |
|  | Height (cm) | 172 ± 8^b^  (171, 173) | 170 ± 7^b^  (169, 171) | 176 ± 8^ab^  (174, 177) |
|  | BMI | 26.5 ± 3.9^b^  (26.0, 27.0) | 26.1 ± 3.6  (25.5, 26.6) | 27.2 ± 4.3^ab^  (26.3, 28.0) |

^1^Mean ± standard deviation (95% confidence interval).

^2^N may be slightly lower for some groups due to missing data.

Significantly different from South Asians (a) or Females (b), P<0.05.
