# Supplementary figures and images for: Low 25-hydroxyvitamin D levels are more prevalent in Canadians of South Asian than European ancestry inhabiting the National Capital Region of Canada
Source: PLoS One. 2018 Dec 12;13(12):e0207429. doi: 10.1371/journal.pone.0207429 (PMC6291105; doi:10.1371/journal.pone.0207429)

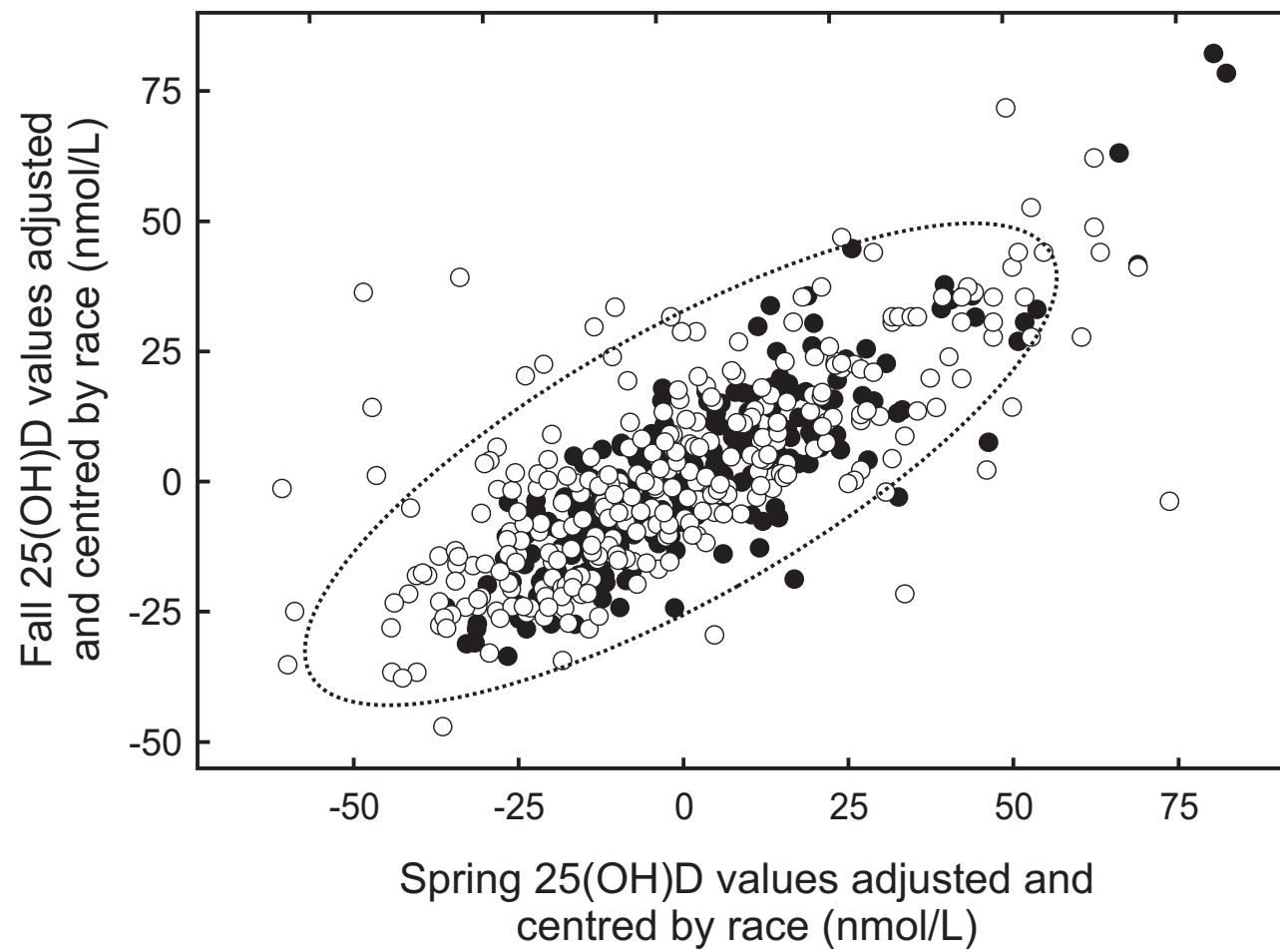

Supplement: S1 Fig — Data includes only participants who attended both spring and fall collections. The relationship between spring and fall values was assessed by partial Pearson correlation analysis with race as the other variable. The 95% prediction ellipse is shown. (PDF) [file pone.0207429.s002.pdf]

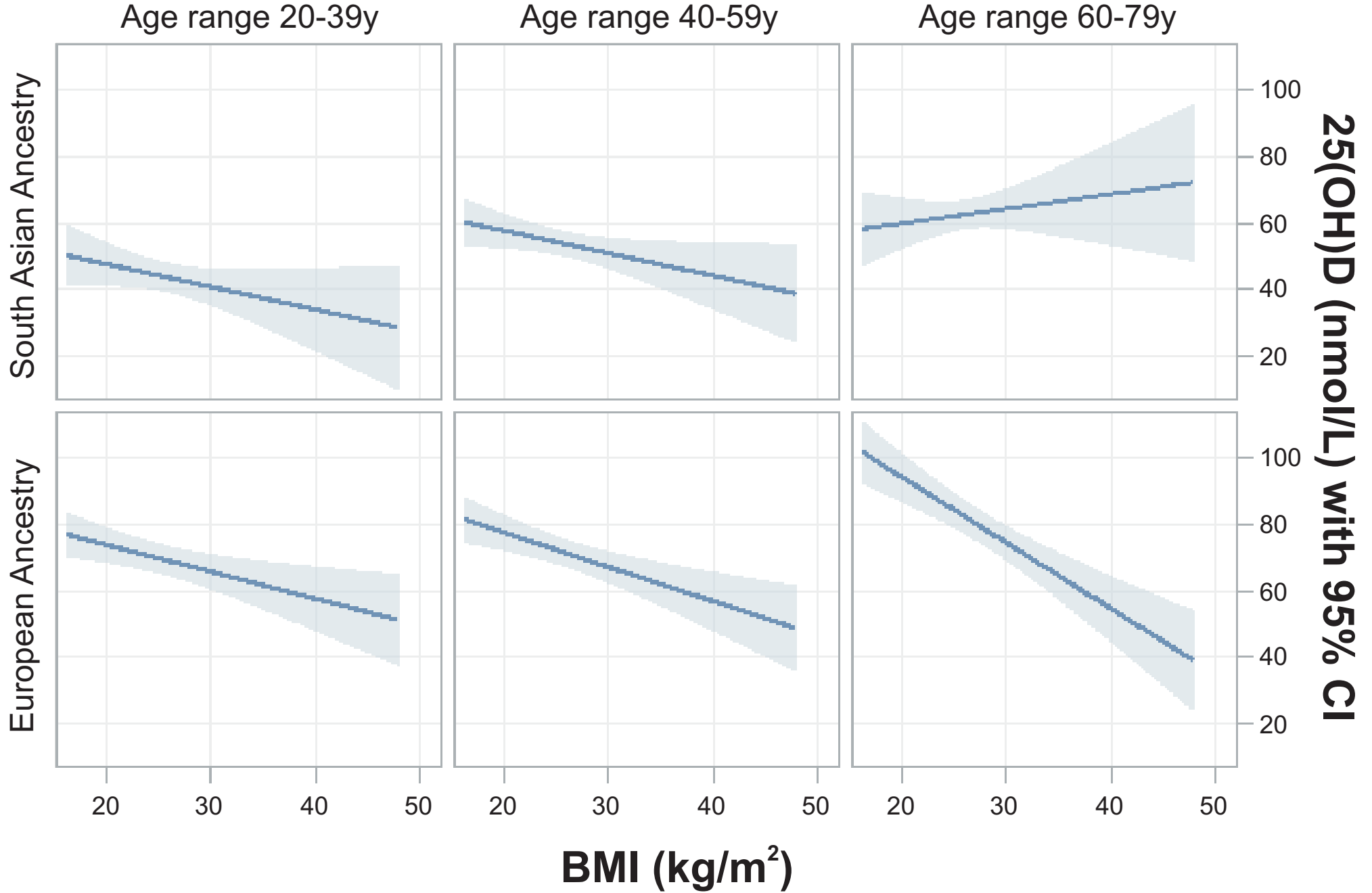

Supplement: S2 Fig — Solid line represents regression of least square means obtained from the PROC MIXED model with shaded area representing the 95% confidence limits. (PDF) [file pone.0207429.s003.pdf]
